# Validation of a food frequency questionnaire as a tool for assessing dietary intake in cardiovascular disease research and surveillance in Bangladesh

**DOI:** 10.1186/s12937-020-00563-7

**Published:** 2020-05-14

**Authors:** Shirin Jahan Mumu, Dafna Merom, Liaquat Ali, Paul P. Fahey, Israt Hossain, A. K. M. Fazlur Rahman, Margaret Allman-Farinelli

**Affiliations:** 1 School of Health Science, Western Sydney University, Sydney, Australia; 2 Dept of Epidemiology, Bangladesh University of Health Sciences (BUHS), Dhaka, 1216 Bangladesh; 3 Pothikrit Centre for Health Studies, Dhaka, 1000 Bangladesh; 4 Dept of Biochemistry & Cell Biology, BUHS, Dhaka, 1216 Bangladesh; 5 School of Life and Environmental Sciences, The University of Sydney, Sydney, Australia

**Keywords:** Food frequency questionnaire, Validity, Dietary assessment, Nutritional epidemiology, Bangladesh

## Abstract

**Background:**

Cardiovascular disease (CVD) has emerged as a major public health concern in Bangladesh. Diet is an established risk factor for CVD but a tool to assess dietary intake in Bangladesh is lacking. This study aimed to validate a food frequency questionnaire (FFQ) using the 24-h dietary recall method and corresponding nutritional biological markers among rural and urban populations of Bangladesh.

**Method:**

Participants of both genders aged 18–60 years were included in the analysis (total *n* = 146, rural *n* = 94 and urban *n* = 52). Two FFQs of 166 items were administered three-months apart, during which time three 24-h dietary recalls were also completed. Participants were asked to recall their frequency of consumption over the preceding 3 months. Urine and blood samples were collected for comparison between FFQ-estimates of nutrients and their corresponding biomarkers. Methods were compared using unadjusted, energy-adjusted, de-attenuated correlation coefficients, 95% limits of agreement (LOA) and quartile classification.

**Results:**

Fair to moderate agreement for ranking energy, macro and micronutrients into quartiles was observed (weighted *k* value ranged from 0.22 to 0.58; *p* < 0.001 for unadjusted data) except for vitamin D (weighted *k −* 0.05) and zinc (weighted *k* 0.09). Correlation coefficients of crude energy, macronutrients and common micronutrients including vitamin E, thiamine, riboflavin, niacin, pyridoxine, folate, iron, magnesium, phosphorus, potassium, and sodium were moderately good, ranging from 0.42 to 0.78; *p* < 0.001 but only fair for vitamin A, β carotene and calcium (0.31 to 0.38; *p* < 0.001) and poor for vitamin D and zinc (0.02 and 0.16; *p = ns*, respectively). Energy-adjusted correlations were generally lower except for fat and vitamin E, and in range of − 0.017 (for calcium) to 0.686 (for fat). De-attenuated correlations were higher than unadjusted and energy- adjusted, and significant for all nutrients except for vitamin D (0.017) to 0.801 (for carbohydrate). The Bland Altman tests demonstrated that most of the coefficients were positive which indicated that FFQ provided a greater overestimation at higher intakes. More than one in three participants appeared to overestimate their food consumption based on the ratio of energy intake to basal metabolic rate cut points suggested by Goldberg. Absolute intake of macronutrients was 1.5 times higher and for micronutrients it ranged from 1.07 (sodium) to 26 times (Zinc). FFQ estimates correlated well for sodium (0.32; *p* < 0.001), and vitamin D (0.20; *p =* 0.017) with their corresponding biomarkers and iron (0.25; *p* = 0.003) with serum ferritin for unadjusted data. Folate, iron (with haemoglobin) and total protein showed inverse association; and fat and potassium showed poor correlation with their corresponding biomarkers for unadjusted data. However, folate showed significant positive correlation (0.189; *p* = 0.025) with biomarker after energy adjustment.

**Conclusion:**

Although FFQ showed overestimation for absolute intake in comparison with 24-h recalls, the validation study demonstrated acceptable agreement for ranking dietary intakes from FFQ with 24-h recall methods and some biomarkers and therefore could be considered as a tool to measure dietary intake for research and CVD risk factors surveillance in Bangladesh. The instrument may not be appropriate for monitoring population adherence to recommended intakes because of the overestimation.

## Background

Cardiovascular disease (CVD) is the leading cause of morbidity and mortality worldwide accounting for 31% of all deaths globally [1, 2] of which > 75% occur in low- and middle-income countries [3]. In Bangladesh, CVD has emerged as an important public health problem with 27% of all deaths attributed to CVD [4]. Ischemic heart disease (IHD) and stroke are now ranked as the top two causes of Years of Life Lost (YLL) in Bangladesh [5]. A recent systematic review of prevalence studies in Bangladesh reported the overall weighted pooled prevalence of CVD was 5%, regardless of gender, region or type of CVD [6]. The prevalence was found to be higher in urban areas (8% [95% CI: 3–14%]) compared to rural areas (2% [95% CI: 1–4%]) [6]. Ischemic heart disease was found to be the most prevalent CVD (21%) whereas stroke was the least prevalent (1%) [6].

Many epidemiological studies have observed that diet plays an important role in the development and therefore, prevention of cardiovascular disease [7, 8]. A diet rich in energy, total fat, saturated fat and sodium but relatively deficient in unsaturated fats, fruits and vegetables has been associated with the progression of CVD risk factors [9]. Recent epidemiological studies have also shown protective associations between intake of B vitamins (folate, vitamin B6 and vitamin B12), vitamin D, antioxidants like β-carotene, vitamin C, and vitamin E, and risk of CVD [10, 11]. Further research is needed to better understand the effect of these nutrients on CVD.

Diet is one of the most complex behaviours to measure and assessing diet is considered the greatest challenge in nutritional epidemiology [12, 13]. Different methods have been designed to assess diet and each of these methods has its own strengths and limitations [12, 14, 15]. The Food Frequency Questionnaire (FFQ) is one of the most common, and is considered a cost-effective and practical dietary assessment method for large samples, such as population based epidemiological studies [13, 14, 16]. However, the FFQ asks individuals to recall information on all types of food from a defined list for a specific time period, including the frequency of consumption and the portion sizes of each item [17]. Such detailed information is subject to random or systematic errors which can lead to biased estimation of the association between diet and disease [13]. Therefore, any FFQ requires validation prior to or as part of dietary research or population monitoring.

Many FFQ have been validated and used in studies in high-income countries. However, few FFQ have been designed and validated in developing countries like Bangladesh [18, 19] where literacy level is low. It is recommended that a FFQ that was developed in one country should not be used in another country unless dietary habits are very similar [20]. Two FFQ validation studies were conducted previously in rural Bangladesh, however, both were developed for a longitudinal study investigating arsenic exposure. The nationwide periodical surveys of non-communicable disease (NCD) risk factors in Bangladesh follow the WHO STEPS strategy where only fruit and vegetable intake have been assessed; lacking information on other elements of dietary intake [21]. Although inadequate fruit and vegetable intake are important risk factors for CVD, long-term surveillance of a broader range of foods is needed to monitor progress toward control of chronic diseases.

Like other Asian countries, Bangladesh is undergoing nutritional transition due to urbanization and globalization [22, 23]. With urbanization or migration to urban areas, there is a marked increase in consumption of fats and sugars and a decrease in the intake of fruit and vegetables. Increased access to and the popularity of fast food may also contribute to poorer diet quality [24]. This study is a precursor to the Migration Study of Bangladesh: a large sibling-comparative study comparing the dietary intake of migrants from rural-to-urban area with their rural siblings. The Migration Study is intended to compare relatively all macro- and micronutrients, associated with development of CVD (such as fat and sodium) and those protective against CVD (such as folate, vitamin E, C, β-carotene). To do so, the FFQ previously designed for the Health Effects of Arsenic Longitudinal Study (HEALS) in Bangladesh [18] was adapted to the context of cardiovascular risk and the list of food items was extended. In the HEALS research, the nutrient intakes using a 39 item FFQ among 189 randomly selected rural residents was compared with two 7-day food diaries (FD) [18]. Focussing on arsenic, the HEALS FFQ was only validated for rural residents. We required a tool validated for both rural and urban residents. As even a subtle change in the design of an FFQ may affect the performance [14], validation of the altered instrument in each population is required. Thus, a validation study was necessary and was performed prior to the data collection phase of the parent study. In this study we compared the FFQ with three 24-h recalls and corresponding nutritional biological markers.

## Methods

### Study population

The validation study sample was recruited from rural (Satia village of Pirganj subdistrict of Thakurgaon District) and urban (Dhaka City) areas where the Migration study of Bangladesh was to be conducted. However, the samples for each study were different. Dhaka is the capital and the largest city in Bangladesh, and the Thakurgaon District is situated at the northern part of Bangladesh (390 km from Dhaka). A total of 162 participants of both genders aged 18–60 years were included in the study. Pregnant women, those with an intellectual disability, or those with any chronic medical condition which required dietary restriction were excluded. The minimum required sample size was calculated to have 80% power to detect a Spearman correlation coefficient of 0.4 [19] or more, between the FFQ items (in grams or calories) and the corresponding 24-h recall, as statistically significant at the α = 0.05 significance level. To validate the FFQ in each region separately, the minimum required sample size was estimated to be 55 urban and 55 rural participants (110 in total).

To select the rural participants, each household (HH) of Satia village was approached starting with the closest house on the left hand side of the main road, and then the next-nearest HH was visited and recruitment continued until the sample size was reached. From each HH one eligible person was selected randomly. If a house was unoccupied or an eligible participant was not present at the time of a visit, the house was revisited later that day or on another day. Urban participants were selected using convenience sampling from faculty and staff of a worksite: Bangladesh University of Health Sciences (BUHS), Dhaka. At BUHS there are twelve distinctive work grades from the highest grade (e.g., professor) to the lowest position (e.g., cleaner). To incorporate all grades into the study, the recruitment methods included email and poster advertisements on the University campus as well as face-to-face conversation to recruit those who were illiterate or have no access to email.

At the first meeting, potential participants received written materials that described the study aims and procedures. Those who provided written consent were recruited to the study. For illiterate participants, research assistants read the materials line-by-line and explained the content in simple words. The research assistant ensured that illiterate participants understood the study properly and made the decision to participate independently. Participants were informed that they could withdraw at any time during the study and were provided with the study telephone number for any later questions or complaints. The study obtained ethics approval from the Western Sydney University Human Research Ethics Committee (HREC # H11145) and the BUHS Ethical Review Committee.

### Study design

**Fig. 1:**
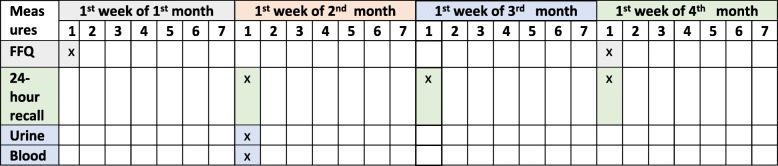
Scheme of the dietary assessments and blood and urine sampling for one participant

Validity was assessed by comparing the energy and nutrient intakes derived from the FFQ against the 24-h recall method and biomarkers. The FFQ and 24-h recall were administered via an interview conducted by trained research assistants, who were equipped with interview manuals and the same reference portion size for standardisation. During field interviews, the nutritionist or field supervisor conducted random visits for quality control. The details on the sequence of administration of the dietary survey methods and blood and urine collection are shown in Fig. 1.

### The food frequency questionnaire

A semi-quantitative FFQ was administered twice, three months apart. The FFQ was based on a questionnaire designed for use in the arsenic study in Bangladesh [18], which was adapted to be used in the migration study of Bangladesh to assess CVD risk. The FFQ used in the HEALS [18] contained 39 food items while the present questionnaire consists of 166 typical food items of rural and urban Bangladeshis, including unique foods of the northern area (Additional File 1). The additional food items were included based on previous studies (unpublished) conducted by BUHS team of nutritionists using the 24-h recall method in Pirganj and Dhaka city. The final food list was generated after checking the availability of foods at local markets and extensive discussion with nutritionists, local research assistants and residents. Food items are listed in the major food groups such as: cereals; pulses & legumes; milk and milk products; meat and fish; eggs; vegetables; fruits; oils and fats; beverages and snacks or fast food. Participants were asked to recall their frequency of consumption over the preceding 3 months. Participants reported the frequency of consumption of each food as never, daily, weekly, monthly, over the entire 3 months and never. To obtain an estimate of portion size, participants were shown common household portions (standard serving size of empty bowl, plate, spoon, ladle, glass) and asked where it was filled to and how many or photographs of different size of same food (i.e. fruits, fish, meat) to pick one. All completed questionnaires were checked by the study nutritionist for accuracy and completeness. As data was collected by trained research assistants, there were relatively few instances of missing data or inconsistency. If any error was found, research assistants contacted the participants by phone for clarification.

### The 24-h dietary recalls

Three 24-h dietary recalls were conducted 1 month apart as a reference measure. As the 24-h recall method collects data on a single day, it cannot describe the usual nutrient intake because of day-to-day variability. However, multiple 24-h recalls provide a reasonable estimate of a person’s usual nutrient intake [15, 25]. Each participant reported all types and amounts of foods and beverages consumed in the previous day. In order to represent all days of the week two surveys were conducted on weekdays and one on the weekend. Specifically, the first 24-h recall was collected on a weekday, and the second or third 24-h recall was performed after a weekend in a random manner. For estimating portion size of consumed foods, participants were encouraged to view commonly used household portions or photographs. The same reference portion sizes were used as for the FFQ. In this study, an average intake from these three recalls was compared to the intake of the last FFQ (which covered the period of three 24-h recalls).

### Biomarkers

A venous blood sample was drawn by a trained phlebotomist after a minimum 8 hours overnight fast. A morning spot-urine was also collected. Venous blood (~ 8 ml) was obtained by venipuncture following standard procedures and the urine sample was collected (10 ml) in a sterile container. Blood samples were collected in a plain tube (~ 8 cc), allowed to clot for 30 min and then serum was separated by centrifugation for 10 min at 3000 rpm. After that, three aliquots of at least 600 μl of serum were collected. For urban participants the blood sample was collected at the BUHS Lab and rural participants were invited to attend a camp for blood and urine sample collection. Samples from rural participants were transferred to the core laboratory at BUHS in a box containing dry ice to maintain a suitable temperature. All samples were preserved in a freezer (− 70 C) until laboratory assays were carried out. All tests were performed at the BUHS laboratory.

The laboratory applied strict quality control techniques and measured the following biomarkers: serum folates (ng/ml), 25-OH vitamin D (ng/ml), ferritin (ng/ml), triglyceride (mg/dl) and sodium (mmol/L), potassium (mmol/L) and creatinine (mg/dl) in urine. Whole blood (~ 2 ml) was taken in a 2 mg/ml EDTA vial for measurement of complete blood count (CBC), measured by flow cytometry method. Serum triglyceride was measured by the GPO-PAP method/ TGL Flex reagent cartridge, (Cat No: DF 69A) and serum [25(OH)D] (Cat No: EIA-5396) and ferritin (Cat No: EIA 1872) were measured using ELISA kits [DRG Instruments GmbH, Germany (Thermo Scientific Multiskan® FC- Filter-based microplate photometer/Finland)]. Serum folate (Cat No: L2KFO2, 200 tests) was measured using chemiluminescence immunoassay (CLIA) (Immulite® 2000 / Siemens, USA). Urine samples were used for urine R/M/E analysis by microscopic examinations, urinary creatinine by CRE2 method/ CRE Flex reagent cartridge, Cat No: DF 33B, and urinary electrolytes by NOVA4 biomedical analyzer.

### Calculation of nutrient intake

The Food Composition Table (FCT) of Bangladesh [26] was used to derive nutrient and energy estimates from dietary data of 122 of the 166 food items collected. Food composition data from other sources [27–31] were used when food items were not available in this FCT. If nutrient values of cooked local foods (30 food items) were unavailable in FCTs, we obtained weighed recipes and a group of nutritionists, including the first author, calculated the nutrient values of those foods. As most of the data in the FCTs are raw food, yield factor was used for cooked food items to convert the quantity of cooked food into raw quantity. Yield factors^1^ were either taken from the FCT of Bangladesh or calculated by weighing before and after cooking following a standard recipe. Estimates of grams of food consumed per day were calculated by multiplying the frequency of consumption of food items by the portion size. This was then converted into daily nutrient intake by using FCTs. For a given nutrient, intakes of all food items were then summed to obtain the total nutrient intake for each individual. Specifically, nutrient estimates were calculated as:

Daily nutrient intake = Σ [(frequency of consumption of a food per day) X (portion size of that food) X (amount of that nutrient in 100 g)].

### Statistical analysis

Mean (±SD) and median with 25th and 75th percentiles were calculated for energy and nutrients assessed by FFQs and the three 24-h recall. The difference between two FFQs were tested by paired t-test for energy and macronutrients, and Wilcoxon signed-rank test for micronutrients. We compared the average of three 24-h recalls to the last FFQ (after 3 months). The Goldberg cut-off values were applied to assess under and over reporting, based on physical activity level (PAL) and compared with the ratio of EI to basal metabolic rate (BMR) [32]. PAL of each participants was investigated by Global Physical Activity Questionnaire (GPAQ) and appropriate PAL value for each participant was assigned accordingly. BMR was estimated using the Schofield equations for adult based on age, gender, height and weight.

Correlations between the two methods were measured using Spearman’s Rank Correlation Coefficient (or Pearson’s Correlation for normally distributed data) for unadjusted; energy-adjusted; age, gender, place adjusted; age, gender, place & energy-adjusted; and unadjusted de-attenuated data. Comparisons were made for the total sample, by gender and place of residency. Energy-adjusted estimates of nutrient intake were obtained by the residual method [33]. De-attenuated correlation was calculated to remove within-person variability in 24-h recalls using this formula: ${r}_t={r}_0\sqrt{1+r/n}$. Here, r_0_ is the observed correlation between FFQ and 24-h recalls, where r is the ratio of intra- and inter-subject variation measured from the three 24-h recalls and n is the number of days of dietary recalls (*n* = 3) [34]. Further, non-parametric partial correlation was used to calculate an age, gender and place adjusted estimate of the correlation. Unadjusted and energy-adjusted correlation was computed between estimates of nutrient intake derived from the FFQ and the corresponding biomarkers. We have presented correlations for the total sample and for the subgroups urban residents and rural residents; and males and females separately. Significance difference of correlation for urban-rural and male-female were tested against each other by Fisher r-to-z transformation. Furthermore, we categorized the distribution of unadjusted and energy-adjusted nutrient intakes into quartiles and then used weighted kappa to assess the agreement between FFQ and 24-h recall. The proportion of subjects categorized in the same quartile by both methods (agreement), in contiguous quartiles (adjacent agreement), and in opposite and/or one quartile apart (disagreement) were estimated. To assess the agreement between two methods the Bland-Altman method was used; first the difference in estimated intake between two methods (FFQ and 24-h recall) were plotted against the average of the estimated intake of these measures [(FFQ + 24-h)/2]. The 95% limits of agreements (LOA) (mean ± 1.96 SD) were calculated for visual illustration of the range of agreement between the two methods. Second, the slope coefficient from the linear regression was calculated for each nutrient. The dependent variable was the difference between dietary intake instruments and the independent variable was the average of two methods. Thus, the slope coefficient estimates the degree of over-or-under estimation over the level of intake.

To interpret the kappa statistic the following standards were used: 0–0.20 = poor; 0.21–0.40 = fair; 0.41–0.60 = moderate/acceptable; 0.61–0.80 = substantial; 0.81–1.0 = near perfect [35]. Socioeconomic classifications were made according to the 2006 per capita Gross National Income (GNI) and according to World Bank (WB) calculations [36]. The groups were: low-income, US$ ≤ 905 or Bangladeshi Taka (BDT) ≤ 5360; lower-middle-income, US$ (906–3595) or BDT (5361–21,270); upper-middle-income, US$ (3596–11,115) or BDT (21271–65,761); and high-income, US$ ≥11,116 or BDT ≥65,762. All *p* values presented were two tailed and *p*-values less than 0.05 were considered to provide statistically significant evidence of association. Data was analyzed using SPSS (version 23) statistical software.

## Results

We recruited 162 participants, of those 90% (146 participants, rural *n* = 94 and urban *n* = 52) completed the final FFQ and three 24-h recall method and were included in the validation study. The main reasons for attrition were moving to another place, quitting their job and an unwillingness to participate due to being too busy. The mean age of the validity study participants was 35 (SD ± 9) years. Slightly over half of the participants were female (55%), 20% had no schooling and the majority (88%) were married. Mean (±SD) BMI and waist circumference were 22.68 (±3.33) and 81.73 (±11.96) cm, respectively. Significant differences were noted between the urban and rural populations for age, education, and income group. The rural group had more participants in the age group of > 40 years old than the urban group (32 and 8%, respectively), while the urban group had more participants in the 31–40 years age group (50 and 39%, respectively). A higher proportion of urban residents had completed high-school or attained a university level of education (70 and 47%, respectively). Nearly significant differences were observed for BMI and waist circumference between the rural and urban groups, indicating higher BMI among urban residents (Additional file 2).

Table 1 presents the mean (±SD) intake for energy and macronutrients, and median with 25th and 75th percentiles for micronutrients derived from two FFQ (baseline and 3 months apart) and three 24-h recalls. The two FFQ estimates indicated no difference for macronutrients, sodium, magnesium and iron but for most of the vitamins there were significant differences. Estimates of energy and nutrients by FFQ were all higher than those obtained from 24-h recalls. Using Goldberg cut-off points, 85.6% of reporting for the 24-h recalls was plausible and 3.4% appeared to be over-reporting intakes whereas over-reporting was found for 36% of participants by FFQ and 60% were plausible reporters. It can be seen that while absolute intakes of macronutrients are up to 1.5 times higher by FFQ method, the percentage energy from protein, fat and carbohydrate are similar. The magnitude of difference in micronutrients ranged from 1.07 (sodium) to 26.5 (zinc) times higher by FFQ than 24-h recalls.

**Table 1:** Energy and nutrient intake per day by FFQ and 24-h recall

Nutrients	FFQ- 1 (***n*** = 162)	FFQ- 2 (***n*** = 146)	24-h recall- 1 (***n*** = 151)	24-h recall- 2 (***n*** = 152)	24-h recall- 3 (***n*** = 148)	24-h recall Average (***n*** = 146)
**Mean ± SD**						
**Energy (kcal)**	3525 ± 1307	3668 ± 1560	2400 ± 838	2486 ± 923	2722 ± 1074	2577 ± 786
**Protein (g)**	137 ± 65	144 ± 75	82 ± 33	86 ± 45	98 ± 57	90 ± 31
**(% energy)**	16 ± 4	15 ± 3	14 ± 3	14 ± 4	14 ± 5	14 ± 2
**Fat (g)**	72 ± 31	74 ± 33	55 ± 27	49 ± 27	51 ± 24	51 ± 21
**(% energy)**	19 ± 6	20 ± 8	22 ± 10	20 ± 11	19 ± 10	20 ± 9
**Carbohydrate (g)**	566 ± 234	586 ± 268	385 ± 174	414 ± 192	457 ± 212	427 ± 167
**(% energy)**	65 ± 8	65 ± 8	64 ± 11	66 ± 13	67 ± 11	66 ± 10
**Median (Q1; Q3)**						
**Vitamin A (μg)**	950.90 (575.83; 2235.12)	1360.05 (608.46; 2221.67)	319.01 (92.41; 1437.14	182.90 (40.02; 641.75)	227.95 (29.20; 632.51)	245.02 (91.72; 613.06)
**β carotene (**μg**)**	5326.82 (2993.34; 8607.47)	4770.12 (2016; 10,380.42)	951.21 (85.53; 6599.98)	667.29 (93.44; 3893.51)	2281.37 (161.19; 6667.31)	1180.59 (161.61; 4137.36)
Vitamin D (μg)*	1.32 (0.62; 2.72)	2.01 (0.98; 3.70)	0.13 (0; 1.37)	0 (0; 1.30)	0 (0; 1.37)	0 (0; 0.93)
Vitamin E (mg)*	9.53 (7.36; 12.05)	7.66 (5.64; 9.71)	5.12 (4.26; 7.37)	5.03 (3.89; 6.81)	5.41 (4.22; 6.69)	4.93 (4.17; 6.48)
Vitamin C (mg)*	338.55 (193.07; 517.51)	318.81 (127.78; 502.35)	48.59 (23.49; 100.30)	69.25 (21.67; 136.00)	100.83 (40.97; 188.80)	74.68 (40.19; 138.44)
Thiamine (mg)*	2.06 (1.51; 2.79)	2.32 (1.23; 3.95)	0.80 (0.58; 1.07)	0.92 (0.66; 1.40)	0.99 (0.67; 1.51)	0.86 (0.72; 1.20)
Riboflavin (mg)*	1.75 (1.16; 2.44)	2.00 (1.10; 3.16)	0.76 (0.46; 1.06)	0.73 (0.48; 1.12)	0.87 (0.55; 1.55)	0.78 (0.57; 1.09)
Niacin (mg)*	30.22 (23.70; 47.19)	36.72 (22.04; 56.02)	17.45 (13.01; 22.00)	17.10 (12.86; 24.16)	17.83 (12.60; 25.55)	17.67 (13.19; 21.94)
Pyridoxine (mg)*	2.86 (2.15; 3.99)	3.44 (1.78; 5.36)	1.47 (1.06; 1.87)	1.60 (1.13; 2.31)	1.94 (1.13; 2.95)	1.63 (1.25; 2.23)
Folate (μg)*	465.02 (327.63; 605.31)	723.28 (274.93; 1183.36)	156.24 (102.04; 240.96)	209.45 (126.84; 324.59)	251.31 (131.79; 512.09)	208.18 (144.22; 303.41)
Calcium (mg)*	1153.00 (688.69; 1970.40)	823.90 (488.07; 1288.51)	427.23 (231.06; 815.12)	309.34 (154.17; 679.85)	390.13 (169.16; 747.60)	417.07 (245.42; 649.60)
**Iron (mg)**	21.20 (15.56; 30.14)	20.28 (12.08; 29.77)	11.64 (8.83; 18.01)	11.29 (7.55; 15.33)	12.20 (8.40; 18.81)	11.71 (9.08; 15.41)
**Magnesium (mg)**	599.45 (444.62; 791.89)	541.01 (373.81; 838.58)	343.97 (223.41; 445.03)	368.97 (255.36; 517.58)	412.56 (260.21; 850.46)	377.16 (273.26; 517.55)
Phosphorus (mg)*	1852.25 (1333.25; 2802.22)	1842.62 (1138.26; 2630.94)	1031.98 (772.92; 1423.72)	1032.96 (733.01; 1499.55)	1130.87 (747.31; 1620.75)	1091.30 (787.46; 1419.22)
**Potassium (mg)**	4024.04 (3104.68; 5015.33)	3854.38 (2349.68; 5501.72)	1895.62 (1472.81; 2477.37)	2060.48 (1465.06; 2854.32)	2233.78 (1620.63; 3461.53)	2071.59 (1668.35; 2641.39)
**Sodium (mg)**	807.66 (559.97; 1193.12)	842.97 (421.99; 1372.02)	405.77 (225.98; 713.32)	404.98 (210.65; 704.52)	399.39 (226.04; 771.00)	401.30 (256.70; 666.97)
**Sodium (mg)**^**a**^	7221.55 (5783.86; 9147.38)	7435.54 (5479.35; 9579.75)	6809.13 (5450.99; 8523.87)	6879.05 (5441.08; 8682.33)	6839.14 (5613.07; 8996.42)	6866.07 (5532.86; 8855.34)
Zinc (mg)*	310.01 (114.70; 639.61)	494.12 (208.31; 1117.18)	13.28 (8.61; 23.83)	13.84 (9.01; 176.32	16.16 (10.43; 734.97)	14.24 (10.14; 20.12)

*Results are expressed as mean (±SD) and median (Q1; Q3);*^*a*^included cooking salt; *Significant difference was observed between two FFQs

Tables 2 and 3 shows the correlation coefficients between the final FFQ and average of the three 24-h recalls for the total group and stratified by urban and rural groups, respectively. All unadjusted correlations were statistically significant except for vitamin D and zinc and energy-adjusted correlations were generally lower except for fat and vitamin E, and in range of − 0.017 (for calcium) to 0.686 (for fat). De-attenuated correlations were higher than unadjusted and energy- adjusted, and significant for all nutrients except for vitamin D, with a range of 0.017 (for vitamin D) to 0.801 (for carbohydrate). De-attenuated correlation coefficient greater than 0.7 were for carbohydrate, vitamin C, pyridoxine, magnesium, and phosphorus. After adjusting for socio-demographic variables, the value of unadjusted and energy-adjusted correlation varied and the range were 0.138 to 0.576 and 0.016 to 0.617, respectively.

**Table 2:** Correlation coefficient of energy and nutrients between FFQ and three days of 24-h dietary recall

Energy and Nutrients	Unadjusted^**a**^	Energy adjusted^**b**^	Age, gender, place adjusted^**c**^	Age, gender, place & energy adjusted^**c**^	De-attenuated
**Energy (kcal)**	0.739**	–	0.481**	–	0.769**
**Protein (g)**	0.555**	0.182*	0.490**	0.210*	0.612**
**(% energy)**	0.131	–	–	–	–
**Fat (g)**	0.424**	0.686**	0.576**	0.617**	0.443**
**(% energy)**	0.800**	–	–	–	–
**Carbohydrate (g)**	0.776**	0.530**	0.396**	0.475**	0.801**
**(% energy)**	0.663**	–	–	–	–
**Vitamin A (μg)**	0.384**	0.145	0.147	0.084	0.427**
**β carotene (**μg**)**	0.309**	0.306**	0.204*	0.278**	0.338**
**Vitamin D (**μg**)**	0.015	0.060	0.174*	0.048	0.017
**Vitamin E (mg)**	0.479**	0.635**	0.494**	0.538**	0.488**
**Vitamin C (mg)**	0.399**	0.064	0.186*	0.016	0.706**
**Thiamine (mg)**	0.598**	0.385**	0.471**	0.370**	0.688**
**Riboflavin (mg)**	0.516**	0.142	0.394**	0.101	0.565**
**Niacin (mg)**	0.553**	0.186*	0.434**	0.183*	0.618**
**Pyridoxine (mg)**	0.738**	0.210*	0.388**	0.129	0.786**
**Folate (μg)**	0.537**	0.043	0.243**	0.015	0.588**
**Calcium (mg)**	0.326**	−0.017	0.149	−0.043	0.359**
**Iron (mg)**	0.530**	0.157	0.248**	0.110	0.568**
**Magnesium (mg)**	0.708**	0.118	0.330**	0.064	0.765**
**Phosphorus (mg)**	0.665**	0.070	0.456**	0.047	0.722**
**Potassium (mg)**	0.617**	0.243**	0.468**	0.229**	0.657**
**Sodium (mg)**	0.342**	0.281**	0.410**	0.276**	0.400**
**Sodium (mg)**^**d**^	0.625**	0.551**	0.510**	0.536**	0.628**
**Zinc (mg)**	0.161	0.206*	0.138	0.167*	0.188*

Correlation was performed between Last FFQ & average of 24-h **Correlation is significant at the 0.01 level; *Correlation is significant at the 0.05 level^*a*^*Pearson correlation coefficient used for energy and macronutrient; Spearman rank correlation coefficient was used for micronutrients*^*b*^*Spearman rank correlation coefficient was performed;*^***c***^*Non-parametric partial correlation was performed*^*d*^*included cooking salt*

**Table 3:** Correlation coefficient of energy and nutrients between FFQ and three days of 24-h dietary recall among urban and rural

Energy and Nutrients	Unadjusted^**a**^		Energy adjusted^**b**^		De-attenuated	
	Urban	Rural	Urban	Rural	Urban	Rural
**Energy (kcal)**	0.642**	0.554**	–	–	0.667**	0.589**
**Protein (g)**	0.659**	0.426**	0.093	0.213*	0.739**	0.473**
**(% energy)**	0.467**	0.224*	–		–	
**Fat (g)**	0.558**	0.565**	0.755**	0.521**	0.576**	0.608**
**(% energy)**	0.606**	0.570**	–	–	–	
**Carbohydrate (g)**	0.429**	0.554**	0.642**	0.430**	0.457**	0.582**
**(% energy)**	0.589**	0.436**	–	–	–	
**Vitamin A (μg)**	0.323*	0.148	0.294*	0.093	0.381**	0.164
**β carotene (**μg**)**	0.385**	0.211*	0.414**	0.248*	0.412**	0.233*
**Vitamin D (μg)**	0.482**	0.082	0.279*	−0.053	0.569**	0.099
**Vitamin E (mg)**	0.688**	0.334**	0.657**	0.380**	0.704**	0.343**
**Vitamin C (mg)**	0.306*	0.186	0.233	0.045	0.338*	0.204
**Thiamine (mg)**	0.392**	0.548**	0.240	0.428**	0.455**	0.637**
**Riboflavin (mg)**	0.594**	0.334**	0.515**	0.044	0.682**	0.364**
**Niacin (mg)**	0.581**	0.424**	0.175	0.160	0.663**	0.478**
**Pyridoxine (mg)**	0.555**	0.420**	0.266	0.143	0.593**	0.457**
**Folate (μg)**	0.319*	0.218*	0.369**	−0.012	0.341*	0.240*
**Calcium (mg)**	0.327*	0.143	0.198	−0.088	0.354**	0.159
**Iron (mg)**	0.480**	0.299**	0.184	0.110	0.524**	0.323**
**Magnesium (mg)**	0.648**	0.286**	0.256	0.070	0.708**	0.314**
**Phosphorus (mg)**	0.593**	0.502**	0.178	0.018	0.690**	0.543**
**Potassium (mg)**	0.466**	0.542**	0.147	0.270**	0.496**	0.576**
**Sodium (mg)**	0.612**	0.355**	0.325*	0.234*	0.710**	0.363**
**Sodium (mg)**^**d**^	0.719**	0.361**	0.681**	0.445**	0.722**	0.419**
**Zinc (mg)**	0.300*	0.110	0.051	0.211*	0.338*	0.130

Correlation was performed between Last FFQ & average of 24-h **Correlation is significant at the 0.01 level; *Correlation is significant at the 0.05 level^*a*^*Pearson correlation coefficient used for energy and macronutrient; Spearman rank correlation coefficient was used for micronutrients*^*b*^*Spearman rank correlation coefficient was performed*^*d*^*included cooking salt*

Stratification by place of residency indicated similar correlation for most of the nutrients (unadjusted and de-attenuated) in urban than rural participants except protein, vitamin D, E, riboflavin, magnesium and sodium where significant higher correlation was observed in urban than rural participants (*p* ≤ 0.05). In the case of energy-adjusted correlation, fat (0.755 versus 0.521) and folate (0.369 versus -0.012) also showed significant higher correlation (p ≤ 0.05) in urban than rural individuals, while the value for protein showed lower for urban individuals than rural though non-significant (0.093 versus 0.213). Correlations between nutrient intake derived from the final FFQ and average of three 24-h recalls were also calculated for men and women (Additional File 3). The correlation coefficient for crude data varied from − 0.112 (vitamin D) to 0.676 (pyridoxine) in men and 0.083 (vitamin D) to 0.789 (carbohydrate) in women. In both genders, adjusting for total energy improved the correlation in some nutrients like fat, vitamin D etc. but decreased the value of most nutrients. However, all correlations increased after de-attenuation except vitamin D in men, ranging from 0.180 (zinc) to 0.752 (carbohydrate) in men and 0.096 (vitamin D) to 0.828 (carbohydrate) in women.

Table 4 lists kappa statistics. The weighted κ value for the unadjusted data ranged from − 0.049 (vitamin D) to 0.582 (energy) and were statistically significant for energy and all nutrients except vitamin D and zinc. For the majority of nutrients, the weighted κ values reduced after energy adjustment, however, increases in values were observed for fat, β carotene, vitamin D and vitamin E. Although κ statistical significance was maintained for macro nutrients, some vitamins (vitamin D, vitamin C and folate) and minerals (calcium, magnesium, phosphorus and zinc) showed non-significant κ statistics. For the unadjusted data, the classification of subjects into same quartile varied from 19% (vitamin D) to 53% (energy) (mean 41%); the exact agreement added to adjacent agreement varied from 61% (vitamin D) to 95% (energy & carbohydrate) (mean 82%); and the disagreement mean was 18%. For energy-adjusted data, the exact agreement mean was 34% and the exact agreement added to adjacent agreement mean was 73%.

**Table 4:** Agreement (weighted k) and cross-classification of quartiles of energy and nutrient intakes

Nutrient	Unadjusted data				Energy-adjusted data			
	Weighted ***k*** (95% CI)	Exact agreement (%)	Exact agreement + Adjacent (%)	Disagreement (%)	Weighted ***k*** (95% CI)	Exact agreement (%)	Exact agreement + Adjacent (%)	Disagreement (%)
**Energy (kcal)**	0.582 (0.495; 0.669)	53.42	95.2	4.79	–			
**Protein (g)**	0.455 (0.354; 0.555)	45.89	87.67	12.33	0.137 (0.020; 0.255)	30.14	69.87	30.14
**Fat (g)**	0.231 (0.116; 0.345)	31.51	76.03	23.97	0.477 (0.381; 0.572)	43.84	91.79	8.22
**Carbohydrate (g)**	0.542 (0.452; 0.632)	49.32	94.53	5.42	0.353 (0.249; 0.458)	36.99	83.62	14.38
**Vitamin A (μg)**	0.263 (0.150; 0.377)	33.56	78.68	21.23	0.135 (0.015; 0.255)	33.56	70.55	29.45
**β carotene (**μg**)**	0.229 (0.107; 0.351)	38.62	73.79	26.21	0.269 (0.151; 0.388)	40.41	76.71	23.29
**Vitamin D (μg)**	−0.049 (−0.163; 0.064)	19.18	60.96	39.04	0.030 (−0.086; 0.147)	25.34	65.75	34.25
**Vitamin E (mg)**	0.27 (0.156; 0.384)	34.97	78.33	21.68	0.377 (0.274; 0.481)	39.04	84.25	15.75
**Vitamin C (mg)**	0.246 (0.134; 0.359)	33.56	75.34	24.66	0.052 (−0.071; 0.175)	28.77	65.76	34.25
**Thiamine (mg)**	0.412 (0.309; 0.515)	41.10	86.99	13.01	0.292 (0.180; 0.403)	36.30	80.14	19.86
**Riboflavin (mg)**	0.346 (0.238; 0.454)	39.04	82.19	17.81	0.150 (0.028; 0.272)	34.25	71.24	28.77
**Niacin (mg)**	0.371 (0.263; 0.480)	41.38	82.07	17.93	0.175 (0.052; 0.298)	34.93	72.6	27.40
**Pyridoxine (mg)**	0.549 (0.460; 0.638)	51.37	93.15	6.85	0.143 (0.023; 0.263)	32.19	69.86	30.14
**Folate (μg)**	0.331 (0.225; 0.438)	38.36	79.46	20.55	0.060 (−0.062; 0.182)	30.14	64.39	35.62
**Calcium (mg)**	0.228 (0.109; 0.346)	36.30	74.66	25.34	0.016 (−1.00; 0.133)	23.40	62.44	33.56
**Iron (mg)**	0.359 (0.246; 0.473)	43.15	80.14	19.86	0.181 (0.059; 0.303)	35.62	72.61	27.40
**Magnesium (mg)**	0.523 (0.427; 0.620)	51.37	90.41	9.59	0.096 (−0.024; 0.216)	32.19	66.44	33.56
**Phosphorus (mg)**	0.447 (0.344; 0.551)	45.89	88.36	11.64	0.055 (− 0.063; 0.173)	28.08	62.33	37.67
**Potassium (mg)**	0.433 (0.334; 0.533)	42.47	88.36	11.64	0.236 (0.119; 0.354)	34.93	77.4	22.60
**Sodium (mg)**^**a**^	0.469 (0.361; 0.571)	51.03	85.51	14.48	0.440 (0.327; 0.552)	50.00	82.88	17.12
**Zinc (mg)**	0.096 (−0.30; 0.223)	31.51	68.5	31.51	0.142 (0.018; 0.267)	32.88	69.87	30.14
**Mean**	0.349	40.62	81.92	18.07	0.191	34.15	73.03	26.68

*Weighted k was performed between Last FFQ & average of 24-h;*^*a*^*included cooking salt*

Table 5 presents the agreement between methods summarized as the mean difference (with 95% LOA) and regression coefficient of the 24-h recall as a predictor of FFQ for each measure of energy and micronutrients. For energy intake, the mean difference between two methods was 1134.78 Kcal with wide limits of agreement (− 1020.16 Kcal to 3289.72 Kcal), with a positive slope coefficient indicating overestimation by FFQ at higher levels of intake. Similar results were observed for other nutrients meaning that the FFQ overestimates intake at higher consumption. The visual inspection of the Bland-Altman plots also indicated a systematic pattern of overestimation at higher intakes and an underestimation at lower intakes of energy and protein intake by FFQ. Bland-Altman plots for the other nutrients showed similar trends but no obvious bias existed for fat, carbohydrate, vitamin A, beta carotene, vitamin D, vitamin E, magnesium, sodium and zinc (Additional file 4).

**Table 5:** Limit of Agreement (LOA) and beta coefficients between FFQ and average of 24-h recall methods

Energy and Nutrients	Mean difference (FFQ- 24-h)	95% LOA lower; upper	β	p
**Energy (kcal)**	1134.78	−1020.16; 3289.72	0.712	< 0.001
**Protein (g)**	55.91	−65.39; 177.21	1.00	< 0.001
**Fat (g)**	21.88	−37.35; 81.11	0.594	< 0.001
**Carbohydrate (g)**	167.69	− 172.68; 508.06	0.485	< 0.001
**Vitamin A (μg)**	932.99	− 1787.16; 3653.14	0.937	< 0.001
**β carotene (μg)**	2368.08	− 8557.61; 13,293.77	0.236	0.03
**Vitamin D (μg)**	1.37	−4.29; 7.03	0.037	0.79
**Vitamin E (mg)**	2.1	−4.52; 8.72	0.494	< 0.001
**Vitamin C (mg)**	223.27	−169.69; 616.23	1.09	< 0.001
**Thiamine (mg)**	1.56	−1.22; 4.34	1.14	< 0.001
**Riboflavin (mg)**	1.19	−1.28; 3.66	0.897	< 0.001
**Niacin (mg)**	20.57	−17.26; 58.39	1.27	< 0.001
**Pyridoxine (mg)**	1.78	−1.36; 4.92	0.955	< 0.001
**Folate (μg)**	475.16	−455.43; 1405.75	1.02	< 0.001
**Calcium (mg)**	402.07	−809.52; 1613.66	0.745	< 0.001
**Iron (mg)**	7.9	−14.09; 29.89	0.607	< 0.001
**Magnesium (mg)**	125.73	− 448.92; 700.38	−0.134	0.13
**Phosphorus (mg)**	741.66	− 707.99; 2191.32	0.90	< 0.001
**Potassium (mg)**	1738.13	− 1450.18; 4926.44	0.96	< 0.001
**Sodium (mg)**	340.72	− 732.52; 1413.96	0.239	0.037
**Sodium (mg)**^**a**^	537.41	− 5020.09; 6094.91	0.282	0.001
**Zinc (mg)**	252.93	− 1703.89; 2209.75	0.118	0.38

Table 6 presents the distribution of mean or median daily concentration of biomarkers and the correlation coefficient for the FFQ estimate and its corresponding biomarker. In the unadjusted correlation, significant positive correlation was found for vitamin D, iron with ferritin and sodium. Negative significant correlation was found for iron with hemoglobin and total protein with urinary creatinine. However, these negative correlations turned to positive after energy adjustment. Vitamin D intake was no longer significantly correlated with blood levels after energy adjustment while the correlation coefficient increased from − 0.053 to 0.189 for folate. For sodium, correlation decreased from 0.322 to 0.227, though was still significantly correlated. We also checked correlation between first 24-h intake and biomarkers and similar findings were observed.

**Table 6:** Level of biomarkers and correlation between FFQ-derived estimates and corresponding biomarkers

FFQ-estimated daily dietary intakes	Biomarkers	Levels of biomarkers		Total		Urban		Rural		Men		Women	
		Mean (±SD)	Median (Q1; Q3)	Unadjusted	Energy adjusted	Unadjusted	Energy adjusted	Unadjusted	Energy adjusted	Unadjusted	Energy adjusted	Unadjusted	Energy adjusted
Vitamin D (μg)	Serum 25 (OH) vitamin- D (ng/ml)	64.13 (±29.98)	58 (42; 82)	0.201*	−0.018	0.095	0.087	−0.012	0.139	−0.086	− 0.242	0.432**	0.193
Vitamin B9 (μg)	Serum folate (ng/ml)	9.73 (±4.77)	7.89 (6.18; 12.80)	−0.053	0.189*	0.323*	0.139	0.169	0.316**	−0.089	0.229	0.020	0.134
Iron (mg)	Serum ferritin (ng/ml)	50.90 (±38.52)	40.30 (25.60; 61.60)	0.247**	0.003	0.163	−0.165	0.060	0.204	0.194	0.255*	0.249*	−0.170
Iron (mg)	Hemoglobin (g/dl)	11.47 (±1.94)	11.20 (9.92; 12.80)	−0.426**	0.082	0.396**	−0.215	0.247*	−0.034	−0.344**	0.209	−0.568**	0.157
Fat (g)	Serum triglyceride (mg/dl)	144.32 (±113.92)	110 (81; 156)	0.162	−0.059	0.103	0.161	0.085	0.017	−0.020	−0.168	0.174	−0.007
Sodium (mg)	Urine sodium (mmol/L)	140.65 (±53.71)	135 (103; 168)	0.322**	0.227**	0.178	0.224	0.245*	0.204	0.380**	0.224	0.329**	0.236*
Potassium (mg)	Urine potassium (mmol/L)	45.56 (±18.37)	45.56 (33; 54)	0.005	0.05	0.131	0.015	0.040	0.035	0.066	−0.003	0.050	0.008
Total Protein (g)	Urine creatinine (mg/dl)	106.97 (±92.95)	78 (36; 136)	−0.186*	0.013	0.058	−0.042	0.063	0.054	−0.124	0.031	−0.230*	0.019

*Results are expressed as mean (±SD) and median (Q1;Q3); Correlation was performed between Last FFQ & biomarkers*

In the subgroup analyses, fair unadjusted correlation was observed for iron with hemoglobin both in urban (0.396) and rural (0.247) areas and the difference between groups were non-significant. While unadjusted correlation for folate was better for urban areas, following energy adjustment, this was reversed to show better agreement in rural individuals. The correlation of sodium intake with urinary sodium concentration is comparable in urban and rural areas (0.224 and 0.204). For the unadjusted correlation, significant positive correlations were found with their biomarkers for Vitamin D, iron and sodium in women and sodium alone in men. After energy adjustment iron was significantly correlated with serum ferritin in men.

## Discussion

The aim of this study was to compare the validity of FFQ with an average of three 24-h recalls and several nutritional biomarkers among urban and rural Bangladeshis. Overestimation of absolute intake of total energy and nutrients were observed by FFQ compared to 24-h recalls, though fair to moderate agreement was found for ranking energy, macro and micronutrients into quartiles indicating the FFQ is good for studying relationships with nutrients intakes. However, at higher levels of intake the Bland Altman tests demonstrated overestimation by the FFQ. In relation to biomarkers, FFQ estimates correlated well for sodium, vitamin D with their corresponding biomarkers and iron with serum ferritin for unadjusted data. Folate, iron (with haemoglobin) and total protein showed inverse association; and fat and potassium showed poor correlation with their corresponding biomarkers for unadjusted data. However, folate showed significant positive correlation with biomarker after energy adjustment.

A major challenge of the validity study is to select a suitable reference method to test the target instrument, as no gold standard exists in dietary intake measurements [34, 37]. While other dietary measurement methods (such as weighed or estimated food records) have been used in validation studies, these were not feasible due to the high level of illiteracy of rural residents and the burden and increased cost involved. It is a limitation that both methods we used rely on memory. However, the 24-h recall has several strengths as it is inexpensive, quick to administer and provides detailed information on food intake. Furthermore, the 24-h recall method requires only short-term memory and can be used for populations in which illiteracy is common [15, 19, 25]. It is considered by some to be more objective than FFQ and its administration does not alter the usual diet as a prospective food record might [15]. In a review article it was reported that about 22% of studies used 24-h recall as the reference method, which was similar to weighed records (25%) [25]. Moreover, in this study we have conducted the 24-h recall for 3 days and on both weekend and weekdays so as to minimise day-to-day variability.

There was overestimation of total energy and nutrient intake by FFQ compared to 24-h recalls, which is in line with previous research [19, 34, 38]. More than one in three participants appeared to overestimate their food consumption based on the ratio of energy intake to basal metabolic rate cut points suggested by Goldberg. It is generally accepted that it is impossible to assess energy intakes using self-report methods but energy adjustment improves estimates of other nutrients [39]. One possible explanation is that when people are asked to recall the frequency of several foods, they tend to overestimate the overall intake [34, 40]. Another possible explanation is that a large number of food items were included in this FFQ: to cover usual and local foods of the city and northern part. Asking more foods might inflate estimates of total intake when summing across foods [19, 41]. There is also a possibility of over-reporting of serving size or frequency of consumption because of biases such as recall and social desirability, which could lead to overestimation of nutrient estimate of FFQ.

Correlation coefficients observed in this study were higher than those found in two earlier studies assessing the validity of FFQs for arsenic against food records in rural areas of Bangladesh [18, 42]. Another comparable study is the Indian Migration Study (IMS) because its objective and study design were quite similar to our parent migration study. The correlation coefficients observed in the IMS FFQ validation study [19] were similar to our study. For example, correlation for fat intake in the IMS was 0.42 which is very similar to our study (0.424).

After energy adjustment, the correlation coefficient in the present study was improved for fat and vitamin E, however, the majority of nutrients showed decreased correlation. When the correlation coefficient increases after energy adjustment, the variability of nutrient intake is related to energy intake. On the other hand, the correlation coefficient decreases if the variability depends on systematic error of under and overestimation [34]. The Bland-Altman plots demonstrated an overestimation at higher intakes and an underestimation at lower intakes. For protein, a linear trend of overestimation was observed in the Bland-Altman plot and a remarkable decline of correlation coefficient (0.659 to 0.093) was observed in urban area after energy adjustment, though rural area showed significant fair correlation. However, fat, which is one of the predictors of CVD, showed better correlation after energy adjustment (0.424 to 0.686).

As demographic variables are always controlled in epidemiological studies as confounders, we also controlled for age, sex and place for unadjusted and energy-adjusted correlation. The adjustment is justified because the between-person variation in dietary intake due to these variables usually increases the observed correlation between methods [37]. However, this study showed fair to moderately significant correlation for most of the nutrients even after adjustment. We also corrected day-to-day within person variation by calculating de-attenuated correlations for energy and nutrients, which were usually higher than their original values. On average, the de-attenuated correlation values were 0.55 for the total sample and higher in the urban (0.55) than rural sample (0.38). The concordance coefficients decreased for weighted κ statistic after categorization of energy and nutrient intake into ordinal level than continuous. However, fair agreement (unadjusted mean κ = 0.349) was observed for most of the nutrients between two methods which was similar to that reported in other validation studies [43, 44]. When allocating nutrient intake into quartiles for the two different methods and looking at cross-classification; subjects were correctly classified in the exact and adjacent quartile with an average of 82% for unadjusted data and 73% for energy-adjusted data. The weighted kappa statistics thus indicate good agreement between methods and these results are comparable to other studies [34, 38, 43].

As all methods of dietary assessment are subject to error [12, 25], we compared FFQ estimates with biological markers. Of the nutrients we considered, folate, sodium and iron estimated by FFQ showed a higher correlation coefficient with the respective biomarker. Folate is one of the protective factors of CVD [45, 46]. In this study, the observed energy-adjusted correlation, around 0.20 for total sample and > 0.30 for the rural subset, suggest a responsiveness of the biomarker to the dietary intake. These correlations were in the range of previous studies [13, 47–50]. Although this study showed significant correlation for total and rural subset, studies often fail to get statistically significant correlations between dietary folate intake estimated by FFQ and serum folate. Possible reasons for these inconsistent findings include information bias, differences in sample sizes and difference in laboratory techniques for folate level estimation [47].

The correlation between sodium intake and urinary sodium for the total sample was fair (0.322). A systematic review on validation of FFQ by sodium biomarker [51] reported the same magnitude of correlation to that obtained in the present study. This systematic review reported that if the FFQ does not include an estimate of discretionary salt use (in cooking or at the table), sodium intake assessed by the FFQ was on average 30% (range 2 to 52%) lower than that measured in 24-h urine collections [51]. Another study on the validation of a FFQ for CVD using biomarkers, also found poor correlation as no question was included on table salt intake [13]. In our study we asked about cooking salt in addition to calculating salt derived from food. The intake of cooking salt was estimated by dividing the monthly usage by the number of family members. When we run our analysis without including cooking salt, the correlation with sodium was poor, as mentioned in the above review [51]. After including cooking salt, significant and fair correlation with urinary sodium were found. In the sub-group analysis, energy-adjusted correlation with urine sodium showed quite similar in urban and rural residents. However, further analyses revealed that the correlations of urban men and women (0.244 and 0.245) was a bit higher than the rural men and women (0.208 and 0.182). One possible explanation for these results is that in rural areas people sometimes use unpackaged salt, which may reduce the accuracy of reporting cooking salt intake in rural participants. Another reason could be the literacy level; but the small sample size in each educational sub-group precluded any investigation. The correlation observed in this study may be increased if a 24-h urine measure was employed. Although multiple 24-h urine collections, assessed for completeness using a suitable method (such as PABA), is recommended [51], we were unable to do this due to feasibility. As sodium intake is related to CVD risk, further study should be done following this recommendation.

Iron overload and deficiency has been proposed to be a potent risk factor for CVD, by different mechanisms [52, 53]. In this study, a fair, unadjusted correlation (0.247) was observed between dietary iron intake and serum ferritin. This reduced to poor (0.003), after energy-adjustment. When we stratified by gender, the energy-adjusted correlation was fair (r = 0.255; *p* = 0.04) for men and poor for women. A similar finding was observed in another study where energy-adjusted correlation between FFQ intake and serum ferritin was poor (r = 0.007) for women [13]. Further sub-group analysis revealed that energy-adjusted correlation was moderate (r = 0.426) for rural men whereas it was poor for both urban men and women (Data not shown). We also tested correlation with haemoglobin, where negative, fair correlation was found with iron intake for the total sample and after stratifying by residence. However, the correlation became positive but poor after energy adjustment. One explanation for this low correlation could be that iron absorption and storage depends on various factors, which were not measured here, such as bioavailability of heme and non-heme iron, interaction with absorption inhibitors and enhancers, infection or inflammation and physiological (menstruation, hookworm) or non-physiological (blood donation) iron loss [13, 37].

Recent epidemiologic studies have demonstrated association between vitamin D insufficiency and the risk of CVD [54]. Although in the unadjusted correlation, vitamin D showed poor (0.201) but significant correlation, it decreased after energy adjustment. The reason for the low correlation might be that plasma vitamin D concentration is influenced by not only diet but also exposure to sunlight, which acts as a confounder [13, 37]. Correlation between protein and potassium intake with their corresponding biomarker showed inverse and poor correlation, respectively, which was not surprising as we did not use recovery biomarkers.

Ideally, in the validation of dietary methods study, recovery biomarkers such as doubly labelled water for energy and markers of potassium, sodium and nitrogen in 24-h urine for potassium, sodium and protein intake would be used [13, 37, 55]. Although recovery biomarkers are considered the gold standard, the expense, availability of these biomarkers and technical expertise required precluded their use in this study.

Other limitations of this study include that we did not consider nutrient retention factors for cooked foods and data on dietary supplements was not collected. Also, although our reference for recall period is short and therefore cannot be affected by seasonal variations, seasonal availability of some fruits and vegetables may lead to a variance in intake of certain vitamins and minerals during the 3 months. The arsenic study of Bangladesh showed small seasonal variation for total energy, protein and carbohydrate intake but larger variation for vitamin D, beta carotene and vitamin A [42]. Studies run across the full year may be warranted. In the current study, reproducibility results were not reported which could be addressed in further studies. Finally, as our urban sample is selected from a worksite; caution should be applied regarding generalization to all urban residents, although our sampling methods ensured urban residents from all SES were included.

The main strength of this study is that it validated the FFQ against multiple 24-h recall measures and biomarkers, among both rural and urban participants. The previous FFQ used in Bangladesh was validated against one other dietary intake measurement (food diary) without any biomarkers, and only on rural Bangladeshis [18, 42]. The recommendation of the previous FFQ [18] study was to include detailed food lists, which was achieved in the current FFQ. We have expanded the food list with frequently consumed food in Bangladesh and included local foods as well. Another strength of this study is the large sample size, including both genders.

## Conclusion

To the best of our knowledge, this is the first validity study of an FFQ for CVD in Bangladesh using multiple measures of dietary assessment. The FFQ showed overestimation for absolute nutrient intakes in comparison with 24-h recalls. However, this validation study demonstrated overall acceptable agreement for ranking individuals by their dietary intakes from FFQ with 24-h recall methods and some biomarkers like sodium intakes. Hence this FFQ can be used to assess the dietary intake in large-scale, epidemiological research or clinical trials in both rural and urban Bangladesh. We recommend this FFQ be used with caution as a tool to monitor population dietary intake and compliance with nutritional recommendations because of the overestimation. Further effort is required to improve its validity for some micronutrients to be tested with recovery biomarkers.

## Supplementary information

**Additional File 1. List of food items**

**Additional File 2.** Table A1. Characteristics of the study subjects according to rural and urban.

**Additional File 3.** Table A2. Correlation coefficient of energy and nutrients between FFQ and three days of 24-h dietary recall among men and women.

**Additional File 4 Fig. A1**. Bland & Altman plot of energy and macronutrient from FFQ and average of 24-h. **Fig. A2**. Bland & Altman plot of vitamin intake from FFQ and average of 24-h. **Fig. A3**. Bland & Altman plot of mineral intake from FFQ and average of 24-h

## Data Availability

The datasets used and/or analysed during the current study are available from the corresponding author on reasonable request.

## Abbreviations

BUHS: Bangladesh University of Health Sciences
CVD: Cardiovascular Diseases
BMR: Basal Metabolic Rate
CBC: Complete Blood Count
FCT: Food Composition Table
FFQ: Food Frequency Questionnaire
HH: Household
IMS: Indian Migration Study
LOA: Limits of Agreement
NCD: Non-communicable Disease
NPNL: Non-Pregnant Non-Lactating
PABA: Para-aminobenzoic Acid
PAL: Physical Activity Level
USDA: United States Department of Agriculture

## Footnotes

1 Yield factors gives information on weight changes during the preparation of foods, for example, water absorption during cooking of rice or water loss during the preparation of meat.
