# Supplementary material for: Validation of a food frequency questionnaire as a tool for assessing dietary intake in cardiovascular disease research and surveillance in Bangladesh
Source: Nutr J. 2020 May 14;19:42. doi: 10.1186/s12937-020-00563-7 (PMC7227307; doi:10.1186/s12937-020-00563-7)
Supplement: Supplementary file 1 — Additional File 1. List of food items [file 12937_2020_563_MOESM1_ESM.docx]

**Additional File 1**

| **Food items** | | | |
| --- | --- | --- | --- |
| **Cereals & gains** | Ganges river sprat (*Kachki*) | Gourd, bottle (*Lau*) | Biscuit sweet |
| Rice | Prawns (*Gura chingri*) | Cucumber (*Sosha*) | Biscuit toasted |
| Roti/ wraps/ chapati | Giant prwan (*Golda chingri*) | Gourd, ridge (*Jhinga/toroi*) | Noodles |
| Paratha | Walking catfish (*Magur*) | Arum (*Kochu*) | *Papor* |
| Bread | Stinging catfish (*Shing*) | Colocasia (*Kochumukhi*) | Potato chop |
| Polao | Tilapia (*Telapia*) | Plantain (*Kacha kola*) | **Fast Foods** |
| Khichuri | Striped snake-head (*Shol*) | Ladies finger (*Dheros*) | Chicken burger |
| Rice Flaked (*Chira*) | Goby (*Bele*) | Raw jackfruit (*Echor*) | Beef burger |
| Puffed Rice (*Muri*) | Indian Batasi (*Batasi* ) | Jackfruit seeds (*Kathaler bichi*) | Sandwich |
| Semolina (*Suji*) | Catfish (*Pabda*) | Gourd, sponge (*Dhundol*) | Chicken fry |
| Vermicelli (*Semi*) | *Guchi* Fish | Bitter gourd (*Korola*) | Chicken patis |
| Birani/ Tehari | *Chang* Fish | Gourd, teasle (*Kakrol*) | Pizza |
| **Pulses and Legumes** | Bigret | Bean (*Shim*) | French fry |
| Lentils (*Mosur dal*) | Glass carp | Carrot (*Gajor*) | **Sweets** |
| Green Gram (*Mung dal*) | Silver carp | Cow Pea (*Borboti*) | *Rosogolla* |
| Black gram (*Maskalai dal*) | Minar carp | Cauliflower (*Fulkopi*) | *Chamcham* |
| Bengal gram (*Cholar dal*) | Gourami (*Kholshe*) | Cabbage (*Badha kopi*) | *Chanar misti* |
| Peas dried (*Motor dal*) | Bronze feather back (*Foli*) | Tanip (*Shalgom*) | *Jilapi* |
| Grass pea (*Khesari dal*) | Spiny eel fish (*Bain*) | Green Peas (*Motor shooti*) | *Rosmalai* |
| **Milk/ Milk Products** | Spotted snakehead (*Shati/Taki*) | **Fruits** | Ice-cream |
| Whole Milk (cow) | Dry fish (*Shutki chingri*) | Coconut (*Narikel*) | Pudding |
| Skim milk (cow) | Dry fish (*Shutki mola*) | Orange (*Komla*) | *Payesh/Khir* |
| Milk powder | **Green Leafy Vegetables** | Apple (*Apel*) | Plane cake |
| Butter | Indian Spinach (*Pui shak*) | Mango (*Aam*) | *Tel pitha* |
| Cheese | Amaranth (*Lal shak*) | Jack fruit (*Kathal*) | *Gurguria* |
| Curd | Bottle ground (*Lau shak*) | Banana (*Kola*) | *Nun pitha* |
| Milk fat/ ghee | Arum leaves (Kochu shak) | Papaya (*Paka pepe*) | *Vapa pitha* |
| **Meat/ Egg** | Spinach (Palong shak) | Guava (*Peyara*) | *Chitoi pitha* |
| Beef | Water Spinach (*Kolmee shak*) | Lychee (*Lichu*) | *Patisapta* |
| Mutton | Jute plant tops (*Pat shak*) | Grapes (*Angur*) | *Narkel pitha* |
| Chicken | Amaranath leaves (*Data shak*) | Lemon (*Lebu*) | **Beverages** |
| Liver (beef/mutton) | *Lafa shak* | Hog plum (*Amra*) | Tea/coffee |
| Cow/goat brain | *Chiramira shak* | Pomelo (*Jambura*) | Cold drinks/energy drinks |
| Hen Egg | Fern (*Dheki shak*) | Jambolan (*Jam*) | Juice |
| Duck Egg | Potato leaves (*Aloo shak*) | Jujube (*Boroi*) |  |
| Koel Egg | Watercress (*Helecha*) | Emblic (*Amloki*) |  |
| Cow's intestine (*Vuri*) | *Sorisha shak* | Carambola (*Kamranga*) |  |
| **Fish** | **Vegetables** | Pineapple (*Anaros*) |  |
| Hilsha (*Ilish*) | Potato (*Aloo*) | Watermelon (*Tormuj*) |  |
| Dragon fish (*Pangash*) | Sweet potato (*Misti aloo*) | Dates (*Khejur*) |  |
| Rohu (*Ru*i) | Brinjal (*Begun*) | Pomegranate (*Anar*) |  |
| Catla (*Katla*) | Radish (*Mula*) | Malta (*Malta*) |  |
| Boal (*Boal)* | Sweet Pumpkin (*Mishti kumra*) | **Snacks** |  |
| Barb (*Sorpunti*) | Gourd (*Chal kumra*) | *Singara* |  |
| Pool burb (*Puti*) | Folwal (*Potol*) | *Samucha* |  |
| Climbing perch (*K*oi) | Gourd, snack (*Kaitha/chichinga*) | *Puri* |  |
| Day’s mystus (*Tangra*) | Green papaya (*Pepe*) | *Peyaju* |  |
| Mola carplet (*Choto mach*) | Tomato (*Tomato*) | Biscuit salted |  |
