# Supplementary material for: Validation of a food frequency questionnaire as a tool for assessing dietary intake in cardiovascular disease research and surveillance in Bangladesh
Source: Nutr J. 2020 May 14;19:42. doi: 10.1186/s12937-020-00563-7 (PMC7227307; doi:10.1186/s12937-020-00563-7)
Supplement: Supplementary file 2 — Additional File 2. Table A1. Characteristics of the study subjects according to rural and urban. [file 12937_2020_563_MOESM2_ESM.docx]

**Additional File 2**

**Table A1 : Characteristics of the study subjects according to rural and urban**

| **Variables** | | | **Total**  **(n=146)** | | **Rural**  **(n=94)** | | **Urban**  **(n=52)** |  |
| --- | --- | --- | --- | --- | --- | --- | --- | --- |
|  | | | **n (%)** | | **n (%)** | | **n (%)** | ***p*** |
| **Age** | Mean±SD | | 35.37±9.19 | | 37.44±9.98 | | 31.67±6.11 | <0.001 |
|  | ≤30 years | 49 (34) | | 27 (29) | | 22 (42) | | 0.004 |
|  | 31-40 years | 63 (43) | | 37 (39) | | 26 (50) | |  |
|  | ≥41years | 34 (23) | | 30 (32) | | 4 (8) | |  |
| **Gender** | | |  | |  | |  |  |
|  | Male | 66 (45) | | 44 (47) | | 22 (42) | | 0.72 |
|  | Female | 80 (55) | | 50 (53) | | 30 (58) | |  |
| **Marital status** | | |  | |  | |  |  |
|  | Married | 126 (81) | | 82 (87) | | 44 (85) | | 0.63 |
|  | Others | 19 (9) | | 12 (13) | | 8 (15) | |  |
| **Education** | | |  | |  | |  |  |
|  | Illiterate or informal education | 29 (20) | | 21 (23) | | 8 (15) | | <0.001 |
|  | Primary school completed | 34 (23) | | 30 (32) | | 4 (8) | |  |
|  | High school completed | 58 (40) | | 37 (39) | | 21 (40) | |  |
|  | University level | 25 (17) | | 6 (6) | | 19 (37) | |  |
| **Income group** | |  | |  | |  | |  |
|  | Low income | 31 (21) | | 30 (32) | | 1 (2) | |  |
|  | Lower-middle income | 87 (60) | | 62 (66) | | 25 (48) | | <0.001 |
|  | Upper-middle income | 24 (16) | | 2 (2) | | 22 (42) | |  |
|  | High income | 4 (3) | | 0 (0) | | 4 (8) | |  |
| **BMI (Kg/m^2^),** Mean±SD | | 22.68±3.33 | | 22.28±3.49 | | 23.41±2.89 | | 0.05 |
| **Waist circumference (cm),** Mean±SD | | 81.73±11.96 | | 80.32±13.93 | | 84.27±6.54 | | 0.056 |

*Results are expressed as mean±SD and number (%) ;t-test and X^2^ test was performed.*
