# Supplementary material for: Validation of a food frequency questionnaire as a tool for assessing dietary intake in cardiovascular disease research and surveillance in Bangladesh
Source: Nutr J. 2020 May 14;19:42. doi: 10.1186/s12937-020-00563-7 (PMC7227307; doi:10.1186/s12937-020-00563-7)
Supplement: Supplementary file 3 — Additional File 3. Table A2. Correlation coefficient of energy and nutrients between FFQ and three days of 24-h dietary recall among men and women. [file 12937_2020_563_MOESM3_ESM.docx]

**Additional File 3**

| **Energy and Nutrients** | **Unadjusted^a^** | | **Energy adjusted^b^** | | **De-attenuated** | |
| --- | --- | --- | --- | --- | --- | --- |
|  | **Men** | **Women** | **Men** | **Women** | **Men** | **Women** |
| **Energy (kcal)** | 0.670** | 0.783** | - | - | 0.701** | 0.825** |
| **Protein (g)** | 0.444** | 0.638** | 0.223 | 0.177 | 0.480** | 0.731** |
| **Fat (g)** | 0.445** | 0.305** | 0.794** | 0.595** | 0.463** | 0.322** |
| **Carbohydrate (g)** | 0.733** | 0.789** | 0.661** | 0.443** | 0.752** | 0.828** |
| **Vitamin A (μg)** | 0.395** | 0.357** | 0.100 | 0.057 | 0.443** | 0.392** |
| **β carotene (μg)** | 0.209 | 0.390** | 0.164 | 0.438** | 0.230* | 0.424** |
| **Vitamin D (μg)** | -0.112 | 0.083 | 0.139 | 0.083 | -0.139 | 0.096 |
| **Vitamin E (mg)** | 0.603** | 0.357** | 0.727** | 0.519** | 0.615** | 0.362** |
| **Vitamin C (mg)** | 0.469** | 0.342** | 0.010 | -0.009 | 0.514** | 0.380** |
| **Thiamine (mg)** | 0.595** | 0.597** | 0.361** | 0.336** | 0.675** | 0.696** |
| **Riboflavin (mg)** | 0.526** | 0.476** | 0.041 | 0.216 | 0.567** | 0.540** |
| **Niacin (mg)** | 0.529** | 0.554** | 0.192 | 0.197 | 0.596** | 0.624** |
| **Pyrodixine (mg)** | 0.676** | 0.743** | 0.142 | 0.208 | 0.718** | 0.801** |
| **Folate (μg)** | 0.573** | 0.508** | -0.069 | 0.022 | 0.626** | 0.561** |
| **Calcium (mg)** | 0.328** | 0.280* | 0.061 | -0.114 | 0.362** | 0.309* |
| **Iron (mg)** | 0.476** | 0.567** | 0.095 | 0.225* | 0.571** | 0.601** |
| **Magnesium (mg)** | 0.641** | 0.725** | -0.125 | 0.243* | 0.693** | 0.787** |
| **Phosphorus (mg)** | 0.633** | 0.675** | 0.152 | 0.014 | 0.666** | 0.759** |
| **Potassium (mg)** | 0.579** | 0.629** | 0.262* | 0.229* | 0.616** | 0.679** |
| **Sodium (mg)** | 0.409** | 0.276* | 0.339** | 0.268* | 0.464** | 0.335** |
| **Sodium (mg)^c^** | 0.671** | 0.590** | 0.592** | 0.510** | 0.673** | 0.593** |
| **Zinc (mg)** | 0.158 | 0.154 | 0.352** | 0.040 | 0.180 | 0.184 |

**Table A2: Correlation coefficient of energy and nutrients between FFQ and three days of 24-hour dietary recall among men and women**

*Correlation was performed between Last FFQ & average of 24-hour*

***Correlation is significant at the 0.01 level; *Correlation is significant at the 0.05 level*

*^a^ Pearson correlation coefficient used for energy and macronutrient; Spearman rank correlation coefficient was used for micronutrients*

*^b^Spearman rank correlation coefficient was performed*

*^c^included cooking salt*
