# Supplementary material for: Validation of a food frequency questionnaire as a tool for assessing dietary intake in cardiovascular disease research and surveillance in Bangladesh
Source: Nutr J. 2020 May 14;19:42. doi: 10.1186/s12937-020-00563-7 (PMC7227307; doi:10.1186/s12937-020-00563-7)
Supplement: Supplementary file 4 — Additional File 4 Fig. A1. Bland & Altman plot of energy and macronutrient from FFQ and average of 24-h. Fig. A2. Bland & Altman plot of vitamin intake from FFQ and average of 24-h. Fig. A3. Bland & Altman plot of mineral intake from FFQ and average of 24-h [file 12937_2020_563_MOESM4_ESM.zip › Additional file 4 - Figure A3R4.docx]

| 1. Calcium | 1. Iron |
| --- | --- |
| 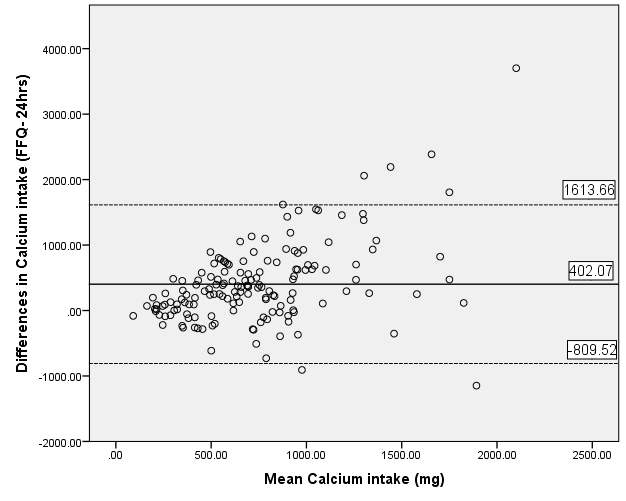 | 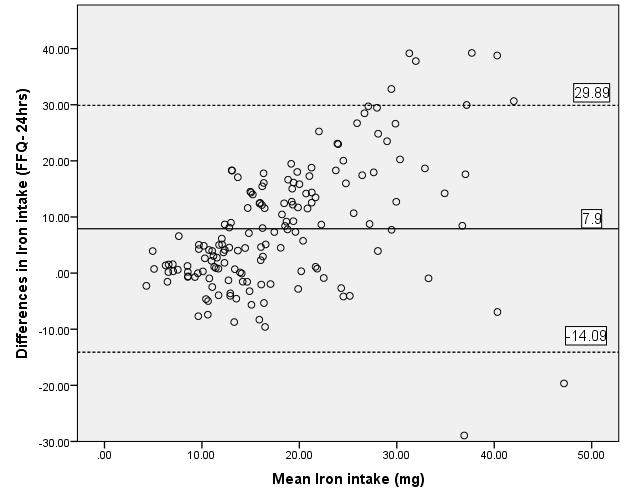 |
| 1. Magnesium | 1. Phosphorus |
| 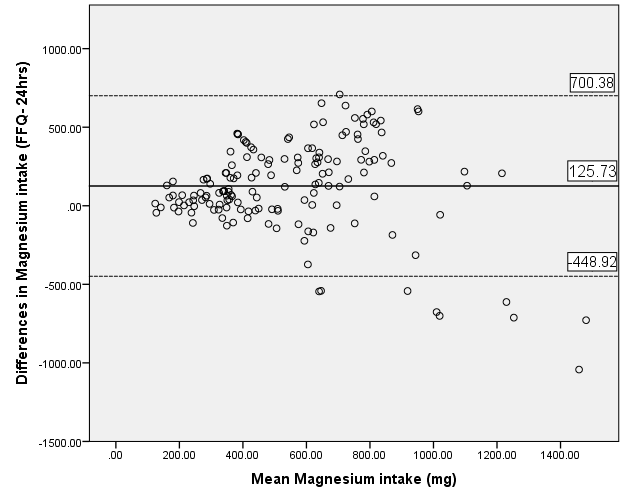 | 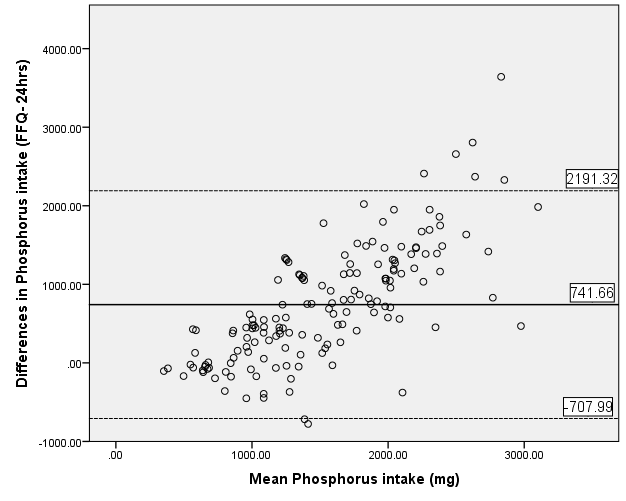 |
| 1. Potassium | 1. Sodium (incl. salt intake) |
| 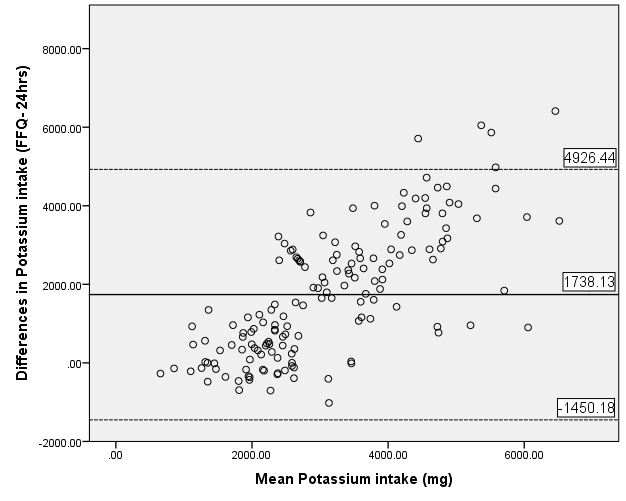 | 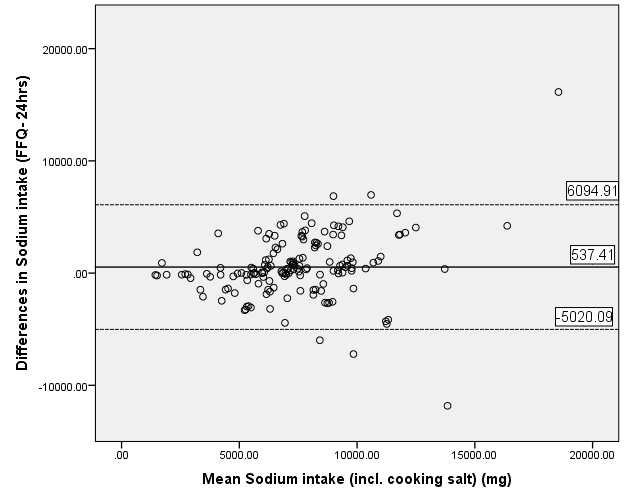 |
| 1. Sodium | 1. Zinc |
| 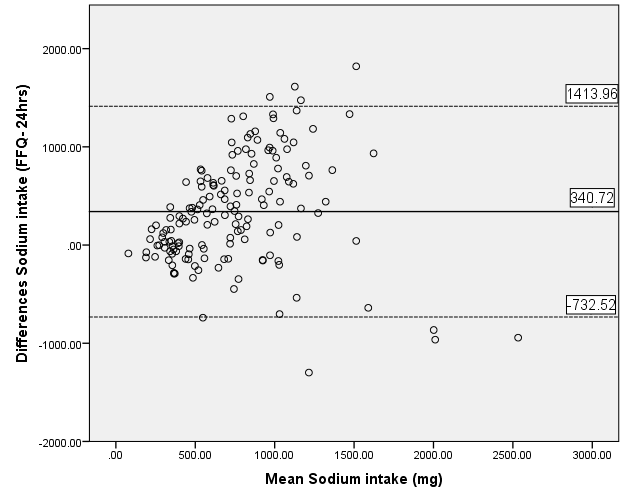 | 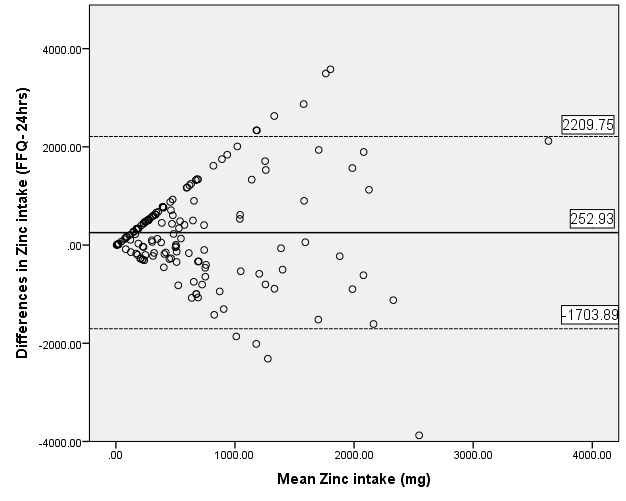 |

**Figure A3: Bland & Altman plot of mineral intake from FFQ and average of 24-hour**
